# Ginseng Oligopeptides Promote Longevity and Enhance Stress Resistance in *Caenorhabditis elegans* via the DAF-16/FOXO Pathway

**DOI:** 10.3390/antiox14121390

**Published:** 2025-11-21

**Authors:** Qian Du, Yiping Zhang, Xiaoyu Guo, Meng Cai, Yong Li, Meihong Xu

**Affiliations:** 1 Department of Nutrition and Food Hygiene, School of Public Health, Peking University, Beijing 100191, China; 2 Key Laboratory of State Administration of Traditional Chinese Medicine for Compatibility Toxicology, Beijing 100191, China; 3 Beijing Key Laboratory of Toxicological Research and Risk Assessment for Food Safety, Peking University, Beijing 100191, China

**Keywords:** ginseng oligopeptides, aging, *Caenorhabditis elegans*, oxidative stress, transcriptomics, DAF-16/FOXO pathway, longevity

## Abstract

**Background:** Ginseng oligopeptides (GOPs), small bioactive peptides with potent antioxidant capacity and high bioavailability, have shown promise in promoting healthy aging; however, their underlying molecular mechanisms remain largely unexplored. **Methods:** Using the model organism *Caenorhabditis elegans* (*C. elegans*), we comprehensively evaluated the anti-aging effects of GOPs on lifespan, locomotion, oxidative stress, and gene expression. Integrated phenotypic assays and transcriptomic analyses were conducted to elucidate GOP-mediated molecular mechanisms. The transgenic strain TJ356 (DAF-16::GFP) and the loss-of-function mutant CF1038 [*daf-16*(mu86)] were employed to functionally validate the role of the DAF-16/FOXO pathway. **Results:** GOP supplementation significantly extended median lifespan by approximately 11.5% and improved age-related locomotion decline in *C. elegans*. Transcriptomic profiling identified 1928 differentially expressed genes (DEGs) enriched in metabolic, antioxidant defense, and longevity-regulating pathways. GOPs upregulated key antioxidant and stress-response genes (*gst-4*, *sod-5*, *mtl-1*) and longevity-related regulators (*daf-16*, *lin-31*, *Y51B9A.9*, and *daf-12*), while downregulating *ins-7*, an insulin-like peptide. Moreover, GOPs enhanced cytochrome P450–related detoxification and vitamin-dependent (retinol, ascorbate, and riboflavin) metabolic pathways, establishing a multidimensional antioxidant defense network. Phenotypic validation confirmed that GOPs markedly reduced reactive oxygen species (ROS) levels and lipofuscin accumulation (*p* < 0.001). Notably, GOPs promoted DAF-16 nuclear translocation in TJ356 worms, whereas the lifespan-extending effects were abolished in CF1038 mutants, highlighting the essential role of DAF-16/FOXO in mediating GOP effects. **Conclusions:** GOPs delay aging in *C. elegans* by activating the DAF-16/FOXO signaling cascade and reinforcing antioxidant networks, thereby maintaining redox and metabolic homeostasis. These findings provide novel mechanistic evidence supporting GOPs as promising dietary antioxidants for promoting healthy aging through modulation of conserved redox and longevity pathways.

## 1. Introduction

Aging represents a progressive decline in physiological integrity, leading to impaired function, increased vulnerability to age-associated diseases, and ultimately reduced healthspan and lifespan [1,2]. In recent decades, population aging has emerged as a global challenge, with functional decline and chronic diseases imposing a growing burden on healthcare systems and quality of life [3,4,5]. Consequently, identifying safe and effective dietary strategies to promote healthy aging and delay age-related functional decline has attracted increasing scientific and public attention [6].

Ginseng (*Panax ginseng*), a traditional medicinal and edible herb, is rich in bioactive constituents such as ginsenosides, polysaccharides, and peptides [7,8]. Ginsenosides, the major pharmacological components, have been shown to extend lifespan, improve oxidative stress resistance, and modulate longevity-related signaling pathways [9]. Ginseng polysaccharides have also been reported to enhance immune function and antioxidant capacity, contributing to the delay of aging processes [10]. In addition to these compounds, ginseng-derived peptides have recently attracted increasing attention due to their small molecular size, high bioavailability, and multi-target biological activity, suggesting a strong potential for anti-aging applications [11]. Our previous studies demonstrated that ginseng oligopeptides (GOPs), small bioactive peptides generated by enzymatic hydrolysis of ginseng proteins, exhibit potent antioxidant, anti-inflammatory, anti-fatigue, and immune-modulatory effects in multiple models [8,12,13,14,15]. However, whether GOPs can directly modulate longevity or aging-related molecular pathways has not been systematically investigated.

*Caenorhabditis elegans* (*C. elegans*), with its short lifespan, well-characterized genetics, and conserved longevity-regulating pathways, has been widely used as a model organism in aging research [16,17]. Among these, the insulin/insulin-like growth factor-1 (IIS) signaling pathway plays a central role in regulating oxidative stress responses, autophagy, and metabolism, thereby influencing both lifespan and healthspan [18]. In *C. elegans*, the key transcription factor DAF-16 (the worm ortholog of human FOXO) functions as the major downstream effector of the IIS pathway, activating antioxidant and stress-response genes such as *gst-4* and *sod-3* to delay the onset of age-related phenotypes [19]. Numerous natural compounds, such as carotenoid esters flavonoids, and bioactive peptides, have been shown to extend lifespan in *C. elegans* via activation of DAF-16/FOXO or related signaling cascades [20,21].

In this study, we systematically evaluated the effects of GOPs on lifespan, locomotor performance, oxidative stress, and age-associated pigment accumulation in *C. elegans*. To elucidate the underlying molecular mechanisms, we performed transcriptomic analyses and further validated the involvement of the DAF-16/FOXO pathway using transgenic and mutant strains. We hypothesized that GOPs delay aging and extend healthspan in *C. elegans* by activating the DAF-16/FOXO signaling pathway, thereby enhancing oxidative stress resistance. Our findings provide novel mechanistic evidence for the anti-aging potential of GOPs and support their application as a functional dietary strategy.

## 2. Methods and Materials

### 2.1. Materials and Reagents

Ginseng oligopeptides (GOPs) were supplied by Jilin TAIGU Biological Engineering Co., Ltd. (Baishan, Jilin, China). The GOPs were derived from the roots of *Panax ginseng* C.A. Meyer through enzymatic hydrolysis, as previously described [8]. GOP powder was dissolved in ultrapure water and sterilized by filtration through a 0.22 μm membrane filter. 5-fluoro-2′-deoxyuridine (FUdR) was purchased from Xinxiyuan Biotechnology Co., Ltd., Beijing, China. The reactive oxygen species (ROS) assay kit was obtained from Beijing Junlibo Biotechnology Co., Ltd., Beijing, China. All other reagents were of analytical grade.

### 2.2. C. elegans Strains and Maintenance

The strains used in this study included wild-type N2, CF1038 [*daf-16*(mu86) I], and TJ356 (zIs356 [*daf-16p*::daf-16a/b::GFP+*rol-6*(su1006)]). All strains were obtained from the *Caenorhabditis* Genetics Center (CGC, Minneapolis, MN, USA). Worms were maintained on nematode growth medium (NGM) plates at 20 °C under standard conditions, using *Escherichia coli* OP50 (*E. coli* OP50) as the food source, following the methods described by Brenner [22]. To eliminate potential interference from bacterial metabolism, the amplified *E. coli* OP50 cultures were inactivated by cobalt-60 (^60^Co) γ-irradiation at a total dose of 10 kGy (dose rate 30 Gy/min, irradiation time 333 min). For GOP treatment, sterilized GOP solutions were mixed with the γ-irradiated *E. coli* OP50 suspensions and evenly spread onto the surface of NGM plates to achieve final GOP concentrations of 0, 100, 200, and 400 μg/mL, with 0 μg/mL serving as the control. The plates were air-dried under sterile conditions before use. Synchronized populations were obtained by bleaching gravid adults with sodium hypochlorite solution, and the eggs were allowed to hatch and develop until the L4 larval stage, following the standard synchronization protocol described by Stiernagle [23].

### 2.3. Lifespan Assays

Synchronized L4-stage larvae (N2 and CF1038) were transferred to NGM plates containing different concentrations of GOPs (0, 100, 200, 400 μg/mL). Twenty nematodes were placed on each plate, and three replicate plates were set for each group. The first day of transfer was designated as day 0. Worms were scored daily under a stereomicroscope (XT-02B, Da Peng, Ningbo, China) for survival, death, and loss. Worms that ruptured, escaped, or displayed vulval bursting were censored. Surviving worms were transferred to fresh GOP-containing NGM plates every two days. Lifespan assays were continued until all worms had died. To prevent progeny from interfering with the counting, 50 μM FUdR was added to the plates.

### 2.4. Locomotion Assays

Synchronized L4-stage worms (N2 and CF1038) were exposed to different concentrations of GOPs on NGM plates. Locomotion assays were performed on unseeded NGM plates at days 3, day 10, and day 16. Worms were transferred to M9 buffer and allowed to acclimate for 30 s, after which head swings and body bends were counted for 30 s under a stereomicroscope. A bend was defined as a change in the direction of the part of the worm corresponding to the posterior bulb of the pharynx. 20 worms were tested in each group.

### 2.5. Lipofuscin (Age Pigment) Accumulation Assay

Synchronized N2 and CF1038 nematodes were cultured on NGM plates containing different concentrations of GOPs for 5 days. Worms were washed with M9 buffer and mounted on 2% agarose pads. 10 μL of 0.4 M sodium azide (NaN_3_) was added to immobilize the worms. Lipofuscin autofluorescence was observed and imaged using a fluorescence microscope (Olympus IX53, Tokyo, Japan) with a 4′,6-diamidino-2-phenylindole (DAPI) filter (excitation 358 nm, emission 461 nm).

All imaging parameters (exposure time, gain, magnification) were kept constant across all groups. At least 30 worms per group were analyzed. The region of interest (ROI) covering the entire worm body was manually defined, and the mean fluorescence intensity was quantified using ImageJ software (Version 1.54p)after background subtraction. The quantified fluorescence values were used for statistical analysis.

### 2.6. ROS Measurement

Intracellular ROS levels were measured using 2′,7′-dichlorodihydrofluorescein diacetate (H_2_DCF-DA). Synchronized N2 and CF1038 worms were exposed to different concentrations of GOPs for 48 h, washed twice with M9 buffer, and incubated with 20 μM H_2_DCF-DA working solution at 20 °C for 45 min in the dark. Worms treated with 100 μM hydrogen peroxide (H_2_O_2_) served as the positive control. After incubation, worms were transferred to 2% agarose pads, immobilized with 0.4 M NaN_3_, and observed under the fluorescence microscope using a fluorescein isothiocyanate (FITC) filter (excitation 488 nm, emission 525 nm), following the same imaging settings as described above. At least 30 worms per group were analyzed. The relative fluorescence intensity was quantified using ImageJ software based on the defined region of interest (ROI) after background subtraction.

### 2.7. DAF-16::GFP Nuclear Localization Assay

The TJ356 transgenic strain expressing DAF-16::GFP was used to evaluate the subcellular localization of DAF-16. Synchronized worms were cultured on NGM plates containing different concentrations of GOPs for 96 h. Worms were washed with M9 buffer, mounted on 2% agarose pads, and anesthetized with 0.4 M NaN_3_. GFP signals were observed using the fluorescence microscope under constant exposure parameters. The subcellular distribution of DAF-16::GFP was categorized into three patterns according to established criteria [24,25]: cytosolic (GFP mainly in the cytoplasm), intermediate (partial accumulation in nuclei but with visible cytoplasmic fluorescence), and nuclear (GFP signals concentrated within nuclei). At least 30 worms per treatment were scored, and the results were expressed as the percentage of worms in each category.

### 2.8. Reverse Transcription Quantitative Polymerase Chain Reaction (RT-qPCR)

L4-stage worms were cultured on NGM plates containing different concentrations of GOPs for 5 days, washed with M9 buffer, and collected. Total RNA was extracted using the Ultrapure RNA Kit (ComWin Biotech, Nanjing, China), and complementary DNA (cDNA) was synthesized from total RNA using the HiFiScript All-in-one RT Master Mix for qPCR (ComWin Biotech, Nanjing, China) and stored at −20 °C.

RT-qPCR was performed using the Takara Real-Time PCR Kit (RR820A, Takara, Kusatsu, Japan) to determine the expression levels of genes related to ageing and oxidative stress. *Ct* values and melting curves were obtained after amplification, and *tba-1* was used as the internal reference gene. Relative gene expression levels were calculated using the 2^−ΔΔ*Ct*^ method, as follows:

M = 2^−ΔΔ*Ct*^

ΔΔ*Ct* = (*Ct*_target_ − *Ct*_tba-1_)_treatment_ − (*Ct*_target_ − *Ct*_tba-1_)_control_

The primer sequences used for RT-qPCR are listed in Table 1.

### 2.9. Transcriptome Analysis

L4-stage worms were cultured on NGM plates containing either 0 (control) or 400 μg/mL GOPs for 5 days, washed with M9 buffer, and collected. Each group included three biological replicates. Total RNA was extracted using the Ultrapure RNA Kit (ComWin Biotech, Nanjing, China). RNA quality was assessed using the Agilent 2100 Bioanalyzer (Agilent Technologies, Santa Clara, CA, USA) and a NanoDrop spectrophotometer (Thermo Fisher Scientific, Wilmington, DE, USA). Only samples with RNA integrity number (RIN) > 7.0 and optical density at 260/280 nm (OD260/280) between 1.8 and 2.2 were used for RNA sequencing (RNA-seq).

RNA-seq library construction and sequencing were performed by Majorbio Bio-Pharm Technology Co., Ltd. (Shanghai, China). Strand-specific mRNA libraries were prepared using the TruSeq Stranded mRNA LT Sample Prep Kit (Illumina, San Diego, CA, USA) and sequenced on the Illumina NovaSeq 6000 platform to generate 150 bp paired-end reads. Each sample yielded approximately 42 million clean reads after filtering. Adapter trimming and quality control were conducted using fastp (v0.23.2). Clean reads were aligned to the *C. elegans* reference genome (WBcel235, Ensembl release 110) with HISAT2 (v2.2.1), and gene-level read counts were obtained using featureCounts (v2.0.3). QC metrics, including GC content, Q20/Q30 scores, and mapping rates, were summarized with MultiQC (v1.13).

Differentially expressed genes (DEGs) were identified using DESeq2 (v1.38.3) in R (v4.3.1), applying |log2FoldChange| ≥ 1 and adjusted *p* < 0.05 as thresholds. Principal component analysis (PCA), volcano plots, and heatmaps were generated to visualize sample clustering and gene-expression patterns. Functional enrichment of DEGs was conducted with ClusterProfiler (v4.8.1) and ReactomePA (v1.44.0) based on Gene Ontology (GO), Kyoto Encyclopedia of Genes and Genomes (KEGG), and Reactome pathways. All raw reads have been deposited in the NCBI Sequence Read Archive (SRA) under accession number PRJNA1357865.

### 2.10. Statistical Analysis

Statistical analyses were performed using GraphPad Prism 10.1.2 (GraphPad Software, San Diego, CA, USA) and R software (version 4.3.1, R Foundation for Statistical Computing, Vienna, Austria). Data are presented as the mean ± standard deviation (SD). Comparisons between groups were performed using one-way or two-way analysis of variance (ANOVA), followed by Tukey’s post hoc test. Survival curves were plotted using the Kaplan–Meier method and compared with the log-rank (Mantel–Cox) test. Subcellular localization distributions of DAF-16::GFP were analyzed using the chi-square test. DEGs from transcriptomic analysis were identified using the criteria |log_2_FC| > 1 and FDR < 0.05. *p* < 0.05 was considered statistically significant.

## 3. Results

### 3.1. GOP Extends Lifespan and Attenuates Age-Associated Decline in Locomotion of C. elegans

**Figure 1:**
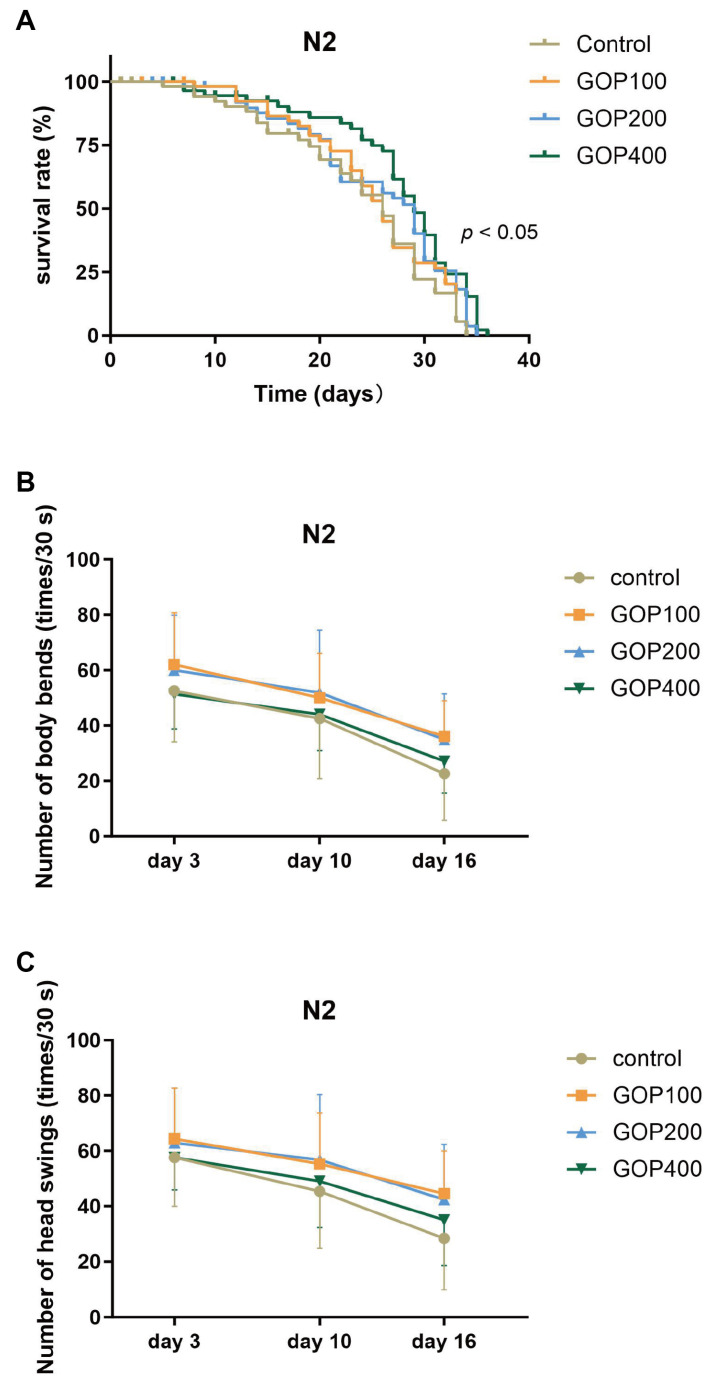
Effects of GOP on lifespan and locomotion of N2 *C. elegans*. (**A**) Kaplan–Meier survival curves of N2 worms treated with different concentrations of GOP. Short vertical ticks on the curves indicate censored individuals. Statistical analysis was performed using the log-rank (Mantel–Cox) test (*p* < 0.05, *n* = 60 per group). Body bend counts (**B**) and head swing counts (**C**) per 30 s measured on days 3, 10, and 16. Data are presented as mean ± SD (*n* = 20 per group per time point). Two-way ANOVA with Tukey’s post hoc test was used for statistical analysis.

Kaplan–Meier survival curves were generated to evaluate the effects of GOP on the lifespan of N2 worms (Figure 1A, Table S1). The log-rank (Mantel–Cox) test revealed a statistically significant difference among groups (*χ^2^* = 7.948, *p* = 0.0471). The trend log-rank analysis further confirmed a dose-dependent extension of lifespan (*χ^2^* = 7.772, *p* = 0.0053). Specifically, the median lifespan of the GOP200 and GOP400 groups was 29 days, representing an extension of approximately 11.5% compared with the control group (26 days).

To further assess the functional benefits of GOP, locomotion assays were performed on days 3, 10, and 16 (Figure 1B,C). A two-way ANOVA revealed a significant main effect of time (*p* < 0.0001), suggesting that locomotor activity of N2 worms declined significantly with increasing age. Compared with the control group, GOP treatment markedly improved locomotion performance (body bends: *p* = 0.0007; head swings: *p* = 0.0021). No significant interaction between time and treatment was observed (*p* > 0.05), indicating that GOP exerted consistent beneficial effects across all time points. Tukey’s post hoc analysis further revealed that, on day 16, body bends and head swings were significantly higher in the GOP100 group compared with the control (*p* < 0.05), and body bends were also significantly higher in the GOP200 group (*p* < 0.05).

### 3.2. GOP Induces Global Transcriptomic Alterations and Activates Longevity-Related Pathways

**Figure 2:**
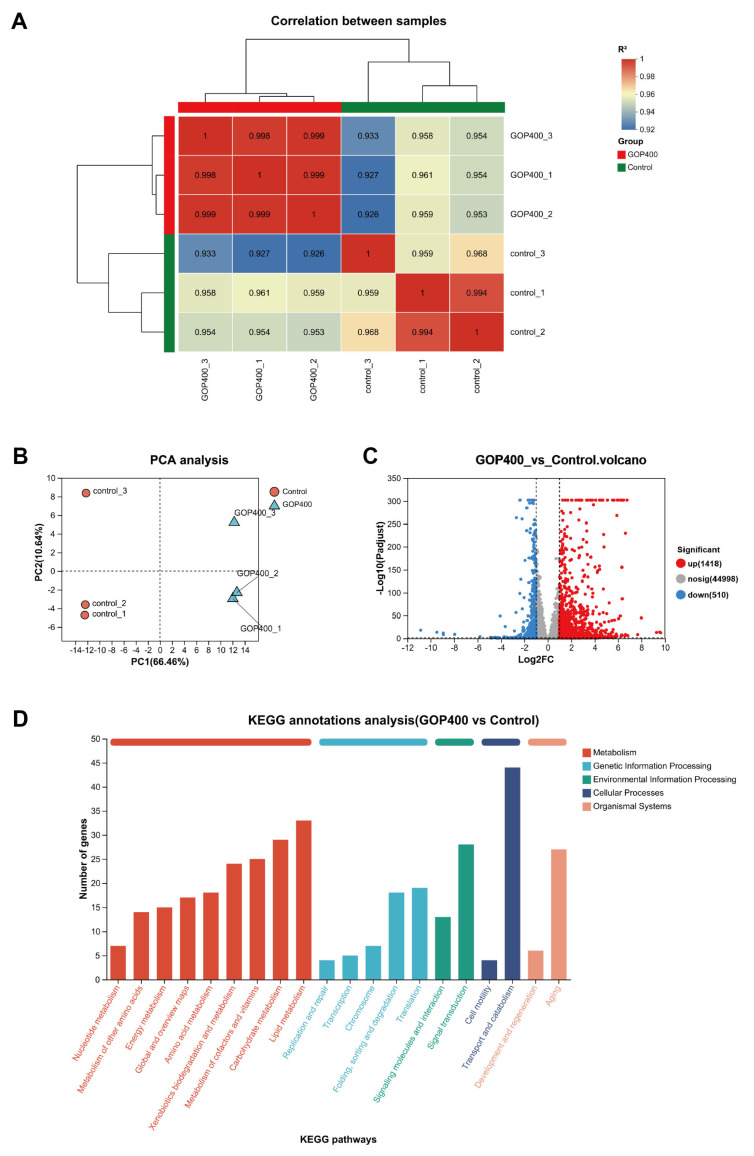
GOPs induced global transcriptomic alterations in *C. elegans*. (**A**) Sample-to-sample correlation heatmap (Pearson *R*^2^) with hierarchical clustering showing high within-group consistency (Control vs. GOP400; *n* = 3 per group). (**B**) PCA plot showing distinct clustering between control and GOP400 groups, with PC1 accounting for 66.46% of the variance. (**C**) Volcano plot of DEGs between GOP400 and control groups. Red dots represent upregulated genes, blue dots represent downregulated genes, and gray dots indicate nonsignificant genes. |log2FC| ≥ 1, FDR < 0.05. (**D**) KEGG functional classification of DEGs.

As shown in Figure 2A, the sample-to-sample correlation heatmap indicated high intra-group consistency and clear separation between the control and GOP400 groups. PCA results revealed a clear separation between the control and GOP400 groups at the transcriptomic level, with PC1 accounting for 66.46% of the total variance (Figure 2B). A total of 1928 DEGs were identified between GOP400 and control worms, including 1418 upregulated and 510 downregulated genes (Figure 2C, Table S2).

Kyoto Encyclopedia of Genes and Genomes (KEGG) annotation showed that DEGs were mainly enriched in metabolic pathways (particularly lipid, carbohydrate, and vitamin metabolism), genetic information processing (transcription, translation, folding, and degradation), and signal transduction pathways, as well as aging pathways (Figure 2D).

**Figure 3:**
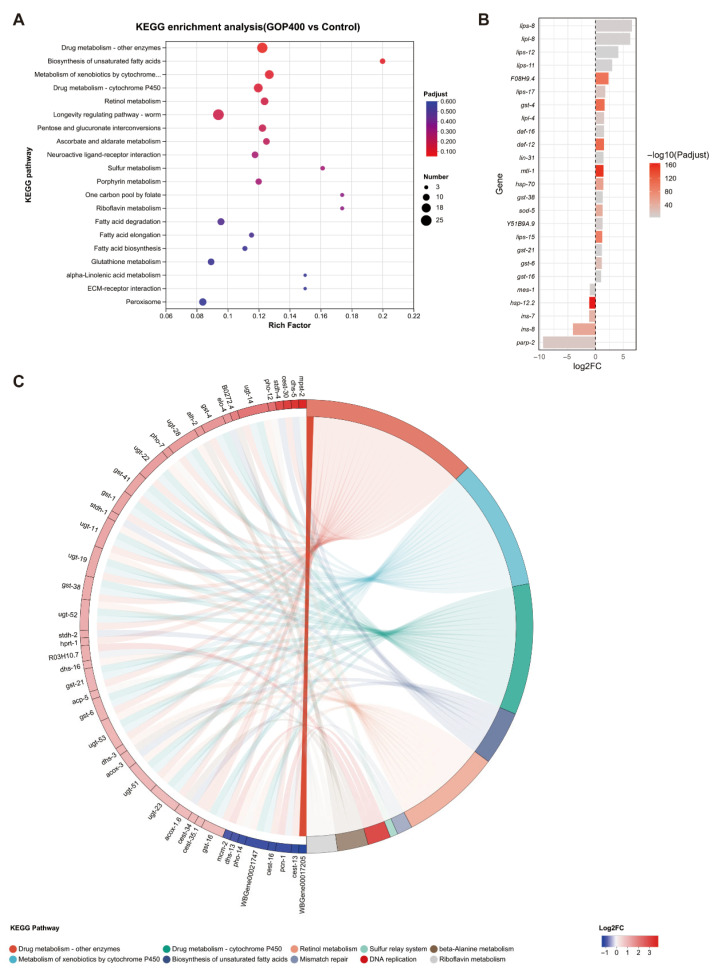
KEGG enrichment of GOP-regulated pathways in *C. elegans*. (**A**) KEGG enrichment bubble plot highlighting significantly enriched pathways. Dot size indicates the number of DEGs, and color represents adjusted *p*-value. (**B**) Expression profile of 25 DEGs enriched in the longevity regulating pathway—worm, ranked by log_2_FC, showing most significantly up- and down-regulated genes. (**C**) Chord diagram illustrating the associations between representative DEGs (left panel) and significantly enriched KEGG pathways (right panel). Each gene is represented as a segment, colored by expression change (red: upregulated; blue: downregulated). Pathways are grouped by functional categories, and chords indicate gene–pathway associations.

KEGG enrichment analysis further revealed significant enrichment of multiple signaling pathways, including the longevity regulating pathway—worm, cytochrome P450-related xenobiotic and drug metabolism pathways, vitamin and antioxidant-related pathways (retinol, ascorbate and aldarate, riboflavin, porphyrin, sulfur, and glutathione metabolism), and lipid metabolism pathways such as biosynthesis of unsaturated fatty acids, as well as fatty acid biosynthesis, elongation, and degradation (Figure 3A).

Further analysis of 25 DEGs significantly enriched in the longevity regulating pathway, ranked by log_2_FC (Figure 3B), revealed that upregulated genes were mainly clustered in multiple lipase family genes (*lips-8*, *lipl-8*, *lips-12*, *lips-11*, *lips-17*, *lipl-4*, *lips-15*), glutathione S-transferase genes (*gst-4*, *gst-38*, *gst-21*, *gst-6*, *gst-16*), superoxide dismutase (*sod-5*), metallothionein (*mtl-1*), and key regulators such as *daf-16*, *lin-31*, *Y51B9A.9*, and *daf-12*. In contrast, the insulin-like peptide gene *ins-7* was downregulated. The enrichment chord plot illustrated the complex interactions between DEGs and key pathways (Figure 3C).

**Figure 4:**
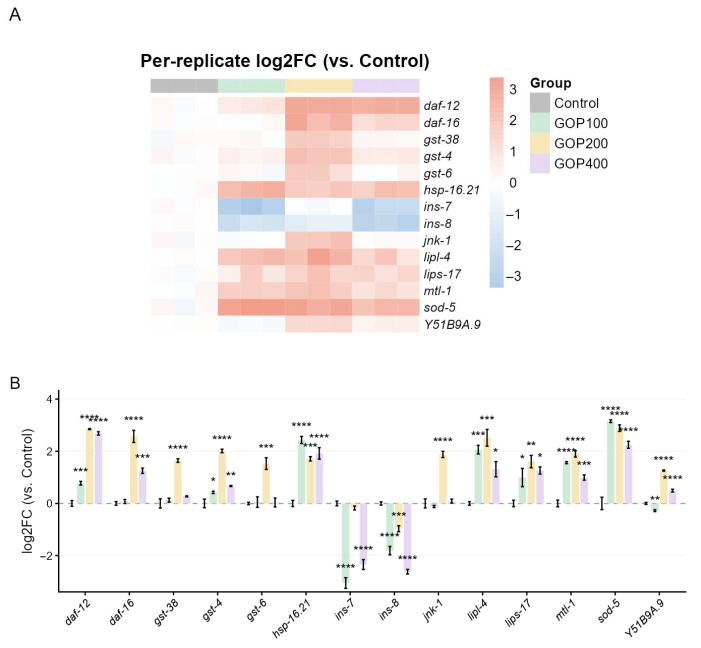
Effects of GOP on the expression of target genes determined by qPCR. (**A**) Heatmap illustrating the log_2_ fold change (vs. Control) for each biological replicate across different GOP concentrations (100, 200, and 400 µg/mL). Each square represents one replicate, and colors indicate upregulation (red) or downregulation (blue). (**B**) Bar graphs showing the mean ± SEM of log_2_ fold change for each gene. Statistical differences among groups were analyzed by one-way ANOVA followed by Tukey’s post hoc test, using the Control group as reference. * *p* < 0.05, ** *p* < 0.01, *** *p* < 0.001, **** *p* < 0.0001.

The qPCR validation further confirmed the transcriptomic findings, showing consistent expression trends for key antioxidant and longevity-associated genes (Figure 4A,B). lipase family genes (*lips-17*, *lipl-4*), antioxidant defense genes (*gst-4*, *gst-6*, *gst-38*, *sod-5*, *mtl-1*), and key regulators (*daf-16*, *daf-12*, *jnk-1*, *Y51B9A.9*, and *hsp-16.21*) were pregulated, while ins-7 and ins-8 were downregulated.

### 3.3. GOP Modulates Pathways Related to Structural Maintenance, Immune Defense, and Cell Cycle Regulation

**Figure 5:**
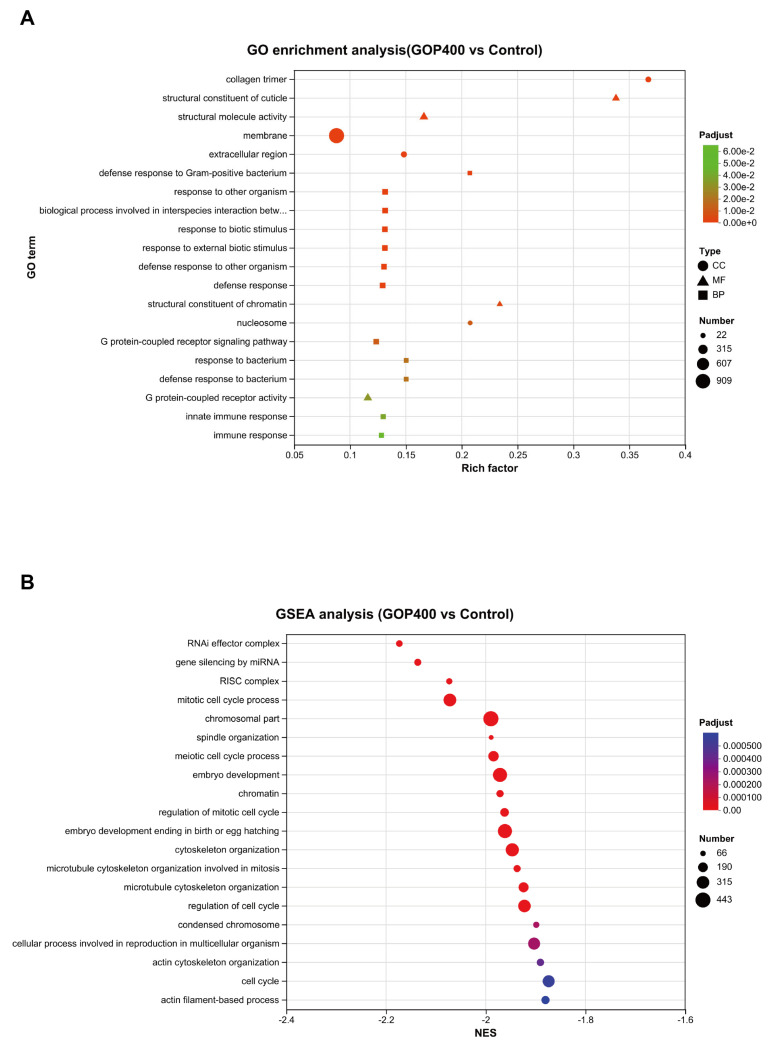
GO enrichment and GSEA analysis of GOP400 vs. control in *C. elegans*. (**A**) GO enrichment bubble plot showing significantly enriched Gene Ontology (GO) terms in GOP400-treated worms. Dot size represents the number of genes enriched, and dot color corresponds to the adjusted *p*-value. Shape denotes the GO category: square = BP (Biological Process), circle = CC (Cellular Component), triangle = MF (Molecular Function). (**B**) GSEA bubble plot displaying significantly enriched GO terms ranked by normalized enrichment score (NES). Dot size indicates the number of genes in each set, and dot color reflects the adjusted *p*-value.

Gene Ontology (GO) functional enrichment analysis revealed that DEGs were significantly enriched in multiple biological processes and molecular functions associated with aging, including structural maintenance (collagen trimer and structural constituent of cuticle), multiple immune responses and defenses, signal transduction regulation (G protein–coupled receptor signaling pathway), and cellular components such as membrane, extracellular region, and nucleosome (Figure 5A).

Further gene set enrichment analysis (GSEA) identified 32 GO pathways that differed significantly between the GOP400 and control groups (adjusted *p* < 0.05). These pathways were mainly associated with RNA interference and gene silencing (RNAi effector complex, gene silencing by miRNA, RISC complex), cell cycle and chromatin-related processes (mitotic cell cycle process, chromatin, spindle organization), and developmental regulation (embryo development and meiotic cell cycle process). These pathways were more active in the control group but were overall downregulated in the GOP-treated group (Figure 5B).

### 3.4. GOP Reduces Intracellular ROS Levels and Lipofuscin Accumulation in C. elegans

**Figure 6:**
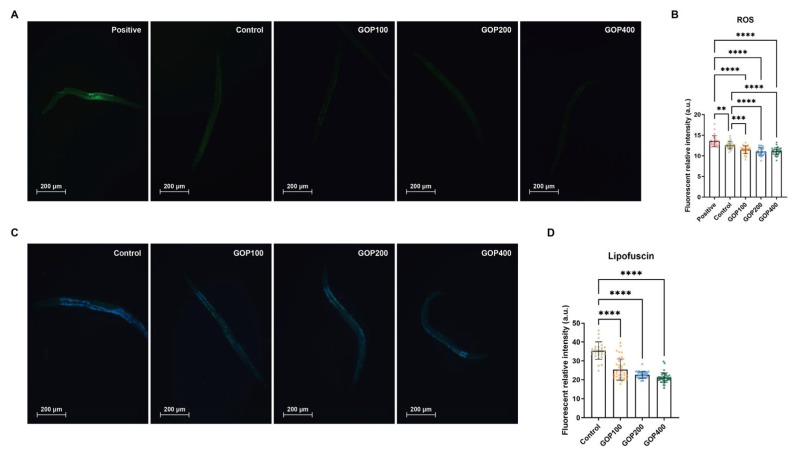
GOPs decrease intracellular ROS levels and lipofuscin accumulation in N2 *C. elegans*. (**A**) Representative fluorescence images of worms stained with H_2_DCF-DA for ROS detection in the positive control (100 μM H_2_O_2_), untreated control, and GOP-treated groups. (**B**) Quantification of ROS relative fluorescence intensity. Data are shown as mean ± SD. One-way ANOVA followed by Tukey’s post hoc test. (**C**) Representative fluorescence images showing lipofuscin autofluorescence in the control and GOP-treated groups. (**D**) Quantification of relative lipofuscin intensity. Data are shown as mean ± SD. One-way ANOVA followed by Tukey’s post hoc test. ** *p* < 0.01, *** *p* < 0.001, **** *p* < 0.0001.

In the ROS assay, one-way ANOVA revealed significant differences in ROS levels among groups (*F* = 79.43, *p* < 0.0001). The ROS level in the 100 μM H_2_O_2_ positive control exhibited markedly higher ROS levels compared with both the untreated control and all GOP-treated groups (*p* < 0.0001), validating successful model induction. Furthermore, the untreated control worms displayed significantly higher ROS levels than GOP-treated worms (*p* < 0.0001), while no significant differences were detected among GOP treatment groups (*p* > 0.05, Figure 6A,B).

In the lipofuscin assay, significant differences in lipofuscin accumulation were also observed among groups (*F* = 9.999, *p* = 0.0006). Tukey’s post hoc test confirmed that the GOP100, GOP200, and GOP400 groups all exhibited significantly reduced lipofuscin levels compared with the control group (*p* = 0.0009, 0.0017, and 0.0129, respectively), whereas no significant differences were observed among GOP-treated groups (*p* > 0.05, Figure 6C,D).

### 3.5. The Anti-Aging Effects of GOP Depend on the DAF-16/FOXO Pathway

**Figure 7:**
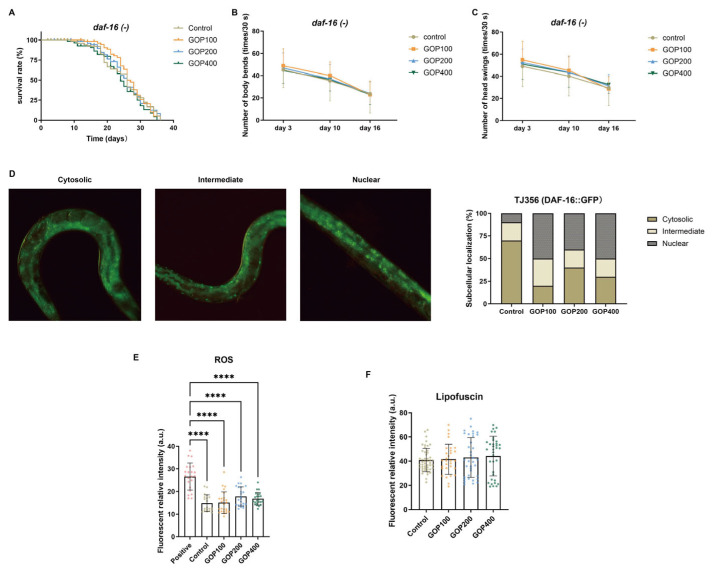
GOP-mediated anti-aging effects depend on DAF-16 activation. (**A**) Kaplan–Meier survival curves of CF1038 [*daf-16*(mu86)] worms. Short vertical ticks on the curves indicate censored individuals. Statistical analysis was performed using the log-rank (Mantel–Cox) test (*p* < 0.05, *n* = 60 per group). Body bend counts (**B**) and head swing counts (**C**) per 30 s in CF1038 worms measured on days 3, 10, and 16. Data are presented as mean ± SD (*n* = 20 per group per time point). Two-way ANOVA with Tukey’s post hoc test was used for statistical analysis. (**D**) Representative fluorescence images of DAF-16::GFP in TJ356 worms and quantification of cytosolic, intermediate, and nuclear localization. Images were captured at 20× magnification. (**E**) Quantification of ROS relative fluorescence intensity in CF1038. (**F**) Quantification of relative lipofuscin intensity in CF1038. Data are shown as mean ± SD (*n* ≥ 20 worms per group). One-way ANOVA followed by Tukey’s post hoc test. **** *p* < 0.0001.

In the *daf-16* null mutant CF1038, GOP treatment did not significantly extend lifespan in any group (*χ^2^* = 2.652, *p* = 0.4484, Figure 7A). Locomotion assays showed that both body bends and head swings of CF1038 worms gradually declined over time (*p* < 0.0001), while no significant differences were observed between the control and GOP-treated groups (*p* > 0.05, Figure 7B,C).

In the TJ356 transgenic strain (DAF-16::GFP), the subcellular distribution of DAF-16 differed significantly among treatments (*χ^2^* = 67.00, *p* < 0.0001). Compared with the control, GOP treatment decreased cytosolic localization and increased nuclear localization, while the proportion of intermediate localization remained largely unchanged (Figure 7D).

To determine whether the antioxidant and anti-aging effects of GOPs depend on DAF-16/FOXO, ROS and lipofuscin levels were examined in the CF1038 mutant strain. As shown in Figure 7E,F, GOP treatment did not significantly reduce intracellular ROS or lipofuscin accumulation compared with the control group.

## 4. Discussion

This study systematically evaluated the anti-aging effects of GOPs in *C. elegans* and elucidated their potential molecular mechanisms through a combination of phenotypic assays and transcriptomic analyses. We demonstrated that GOP supplementation extended lifespan, improved locomotor function, and reduced ROS levels and lipofuscin accumulation. Transcriptomic profiling revealed that GOPs modulated key pathways related to metabolism, detoxification, and longevity, and functional validation indicated that the DAF-16/FOXO pathway played a central role in mediating these effects.

### 4.1. GOPs Exhibit Anti-Aging Effects in C. elegans

We found that GOPs extended the lifespan of N2 worms and alleviated age-related locomotor decline, consistent with previous reports on food-derived bioactive peptides. Imidazole dipeptides from whale meat [26], shark skin peptides [27], sturgeon peptides [28], sea cucumber peptides [29], and rice bran peptides [30] have all been reported to promote healthspan in worms through antioxidant and metabolic mechanisms.

Interestingly, the improvement in locomotion did not exhibit a strict dose-dependent pattern, as the GOP100 group showed the most pronounced effects on body bends and head swings. A similar phenomenon has been reported by Zavagno et al. [31], who found that several compounds enhanced movement at intermediate concentrations but not at higher doses. They attributed this non-linear response to hormetic mechanisms—where mild stress enhances neuromuscular function and resilience, whereas excessive exposure may induce metabolic or oxidative burden. Moreover, movement performance in *C. elegans* is a more sensitive and short-term indicator of physiological function than lifespan, which reflects cumulative systemic regulation [31]. Together, these factors may explain why locomotor improvement peaked at moderate GOP doses, while lifespan extension required higher concentrations.

### 4.2. Transcriptomic and qPCR Evidence Reveals GOP-Mediated Activation of Antioxidant, Metabolic, and Longevity Pathways

To elucidate the molecular mechanisms underlying the anti-aging effects of GOPs, we performed RNA sequencing in *C. elegans*. GOP treatment induced global changes in gene expression, implicating multiple networks associated with metabolic homeostasis, oxidative defense, and longevity regulation. KEGG enrichment highlighted the significant activation of the longevity regulating pathway, suggesting that GOPs act via conserved longevity signaling cascades. DEGs further revealed coordinated activation of core longevity pathways, including *daf-16* (FOXO4), *lin-31* (FOXB2, MAPK transcription factor), *Y51B9A.9* (JNK-like kinase), and *daf-12* (nuclear receptor), alongside the downregulation of *ins-7*, an insulin-related peptide that positively regulates the IIS pathway. Consistent with these findings, qPCR results confirmed that *daf-16* and *daf-12* were both significantly upregulated, suggesting activation of transcriptional regulators that coordinate stress resistance and metabolic adaptation. The InsR-PI3K-AKT-FOXO signaling axis is well recognized as a central regulator of stress resistance and lifespan in *C. elegans* [32]. Our data indicate that GOPs may activate DAF-16/FOXO via the suppression of IIS signaling (through *ins-7*, *ins-8*) while simultaneously stimulating the JNK–MAPK branch (*Y51B9A.9*, *jnk-1)*, thereby promoting oxidative stress resistance, DNA repair, and lipid homeostasis, ultimately contributing to longevity.

In addition to transcriptional regulation, GOP-treated worms exhibited upregulation of canonical antioxidant genes, including *gst-4*, *gst-38*, *gst-6*, *sod-5*, and *mtl-1*. These genes play central roles in neutralizing reactive oxygen species and maintaining intracellular redox equilibrium. Notably, *gst-4*, *sod-5*, *and mtl-1* are well-established downstream targets of DAF-16/FOXO, their concerted upregulation strongly supports the activation of DAF-16–mediated antioxidant defense by GOPs.

Additionally, enrichment of cytochrome P450–related detoxification, vitamin, and antioxidant metabolic pathways indicated that GOPs may establish a multidimensional antioxidant defense network. Cytochrome P450 enzymes (CYPs) are heme-dependent monooxygenases that catalyze oxidative metabolism and detoxification of exogenous compounds, maintaining cellular redox balance [33]. Retinol (vitamin A), ascorbate (vitamin C), and riboflavin (vitamin B_2_) metabolism constitute a vitamin-dependent antioxidant system that scavenges ROS directly or regenerates reduced cofactors such as FAD, FMN, and GSH [34,35,36]. Porphyrin metabolism contributes to heme synthesis, essential for CYP activity and other oxidoreductases involved in detoxification and oxidative metabolism [37]. Moreover, sulfur metabolism provides cysteine and other thiol-containing precursors for glutathione biosynthesis, while the glutathione pathway directly detoxifies H_2_O_2_ and electrophilic species through redox cycling [38,39].

GOPs also significantly enriched unsaturated fatty acid biosynthesis and fatty acid degradation pathways, implying enhanced lipid turnover and energy mobilization that contribute to stress resistance and longevity. Lipid homeostasis has been identified as a central determinant of healthy aging, and active unsaturated fatty acid metabolism is a hallmark of longevity [40]. Upregulation of lipase family genes (*lips-8*, *lipl-8*, *lips-12*, *lips-11*, *lips-17*, *lipl-4*, *lips-15*) suggests the activation of lipolysis and lipophagy, which facilitates energy substrate recycling to sustain antioxidant defenses. This lipid remodeling may help maintain energy–redox balance under stress, an essential factor for healthy lifespan maintenance in *C. elegans*.

GO analysis further identified enrichment of structural maintenance (collagen and cuticle components), immune defense, and GPCR-mediated signaling pathways, potentially supporting improved locomotor performance and stress resilience. Meanwhile, GSEA revealed downregulation of cell cycle and chromatin organization processes, implying that GOPs may reduce excessive proliferation, genomic instability, and energy expenditure, thereby reallocating metabolic resources toward oxidative protection and repair.

**Figure 8:**
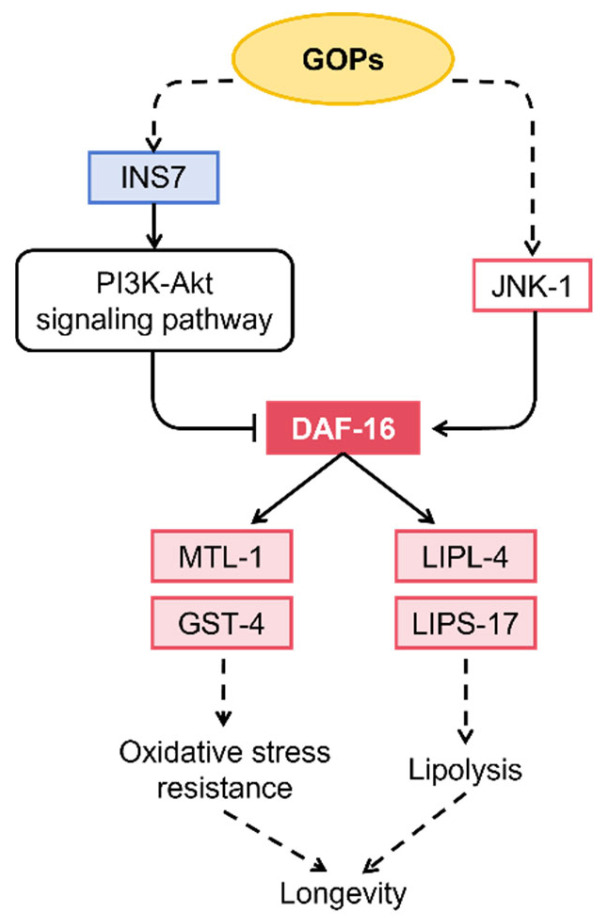
Proposed mechanism of GOP-mediated longevity regulation in *C. elegans*. GOPs may inhibit the insulin-like signaling pathway via suppression of *ins-7* and activation of stress-responsive JNK-1 signaling, thereby promoting DAF-16 nuclear translocation. Activated DAF-16 upregulates antioxidant genes (*gst-4*, *mtl-1*) and lipolytic genes (*lipl-4*, *lips-17*), enhancing oxidative stress resistance and lipid metabolism to extend lifespan. Solid arrows indicate experimentally validated regulatory relationships observed in this study, whereas dashed arrows represent putative pathways inferred from KEGG analysis that have not been experimentally validated. Arrowheads indicate activation, while bar-headed lines indicate inhibition.

Overall, these transcriptomic and qPCR findings suggest that GOPs enhance redox and metabolic homeostasis through dual activation of antioxidant (vitamin-dependent and thiol-based) and longevity signaling pathways (Figure 8), forming a molecular basis for the observed reduction in ROS accumulation and DAF-16 nuclear translocation demonstrated in subsequent phenotypic validation experiments.

### 4.3. GOPs Attenuate Oxidative Stress and Reduce Aging-Associated Biomarkers

ROS accumulation and lipofuscin deposition are well-established molecular and morphological hallmarks of aging [41,42]. In this study, GOP treatment markedly reduced both ROS levels and lipofuscin accumulation in worms, supporting their strong antioxidant and anti-aging capacity. These results are consistent with previous findings showing that peptides derived from *Sepia esculenta* [43], sea cucumber [29], and chicken liver [44] exert similar effects. GOPs may act, at least in part, through the upregulation of enzymatic antioxidant systems such as glutathione S-transferases and superoxide dismutases, thereby reinforcing redox homeostasis and protecting against age-related oxidative damage. The marked reduction in ROS levels and lipofuscin accumulation further substantiates the transcriptomic activation of antioxidant networks, establishing a functional link between GOP-mediated redox regulation and FOXO-driven longevity.

### 4.4. DAF-16/FOXO Signaling Mediates the Longevity Effects of GOPs

DAF-16/FOXO serves as a key transcription factor downstream of IIS pathway and functions as a central hub in the genetic regulation of longevity in *C. elegans* [41,45]. Transcriptomic analysis suggested that GOPs may activate DAF-16/FOXO to promote lifespan extension. Functional validation experiments supported this hypothesis: In the *daf-16* mutant strain CF1038, GOP treatment failed to extend lifespan or improve locomotor performance, whereas in the TJ356 (DAF-16::GFP) strain, it significantly promoted the nuclear translocation of DAF-16. The absence of significant ROS and lipofuscin reduction in CF1038 worms further supports that DAF-16/FOXO is essential for GOP-mediated stress resistance and longevity. These findings provide direct evidence that the anti-aging effects of GOPs are dependent on DAF-16/FOXO activation. Similar mechanisms have been observed with other natural products such as *Anoectochilus* extracts, saffron, and black goji anthocyanins [20,46,47].

Although our data demonstrate that GOP treatment significantly extended lifespan and improved stress resistance in *C. elegans*, the precise mechanisms remain multifactorial. The present findings indicate that the DAF-16/FOXO pathway plays a crucial role, as supported by transcriptomic results and the altered response observed in *daf-16* mutant worms. However, GOP-induced longevity is unlikely to be mediated exclusively through DAF-16. Multiple longevity-related signaling cascades—including SKN-1/Nrf2, HSF-1, and autophagy pathways—are known to interact with DAF-16 to maintain cellular homeostasis under stress conditions. Furthermore, GOPs may directly influence redox balance by modulating antioxidant enzyme activities and metabolic pathways, as suggested by the expression of oxidative stress-related genes. Future studies integrating genetic and biochemical validation will be necessary to delineate the coordinated network through which GOPs exert their anti-aging effects.

### 4.5. Limitations and Perspectives

Despite these promising findings, several limitations remain. First, this study was conducted exclusively in *C. elegans*, whether these effects can be extrapolated to higher organisms or humans requires further validation. Second, GOPs represent a peptide mixture derived from ginseng, and the precise active sequences and structural determinants remain to be identified. Third, although transcriptomic analysis revealed significant enrichment of lipid metabolism pathways, direct experimental evidence confirming GOP-mediated regulation of lipid mobilization or lipid droplet dynamics was not obtained in this study.

Future work should validate the anti-aging effects of GOPs in mammalian models and employ peptidomics to identify the key active peptide sequences. Integration of multi-omics analyses (transcriptomics, metabolomics, and proteomics) and clinical studies will be critical for translating these findings into functional food applications.

## 5. Conclusions

This study systematically demonstrates that GOPs extend lifespan, delay locomotor decline, and reduce ROS and lipofuscin accumulation in C. elegans. These effects are likely mediated through activation of the DAF-16/FOXO pathway, leading to enhanced antioxidant capacity and metabolic homeostasis. To the best of our knowledge, this is the first report to investigate the anti-aging effects of ginseng-derived oligopeptides in C. elegans. Our findings provide molecular evidence supporting GOPs as promising functional dietary components for anti-aging interventions.

## Figures and Tables

**Table 1:** *C. elegans* Oligonucleotide Primer for RT-qPCR.

Gene	Forward (5′ to 3′)	Reverse (5′ to 3′)
*tba-1*	TCAACACTGCCATCGCCGCC	TCCAAGCGAGACCAGGCTTCAG
*gst-4*	ATGCTCGTGCTCTTGCTGAG	CCAAATGGAGTCGTTGGCTT
*gst-6*	GCACGCAAAAATAACACTCCA	CGGTGTCATTTTGTCCAGCA
*gst-38*	TTCAAAGCGGCCGGAAAAAC	AAGATAACGAGCCATCGCGT
*daf-16*	AATTCCTCTCAACAGCAGCAGACC	ACCACCGCCTTGTGACAGATTAAG
*daf-12*	CCTATCAACAAACGTGCGGC	TGTGCACTATTGCCAGATGATG
*mtl-1*	CATGGCTTGCAAGTGTGACTG	TCTCACTGGCCTCCTCACAG
*sod-5*	ATGCCGTTCTTCCACAGGAC	TTCACCTTCGGCTTTCTGGG
*lipl-4*	TGTGTGCAACATGAGAGAATCAA	TGATTTTATTAATTCCGGCGTATCT
*lips-17*	GGGATGGAGTGAAGAATTGGTGT	GGGATGGAGTGAAGAATTGGTGT
*jnk-1*	CGTATCCGTCACATCCAGGTAG	GTCCACGGGTTCCTCGAATG
*Y51B9A.9*	TCGAGTTGGTCACAGAGACTT	TCTTCTCGATTTGCATGTGCG
*ins-7*	GCATGCGAATCGAATACTGAA	GGACAGCACTGTTTTCGAATGA
*ins-8*	ATTGTGCGGAAAGCAAGTCT	TCGCAATATCGACTTTTGTATTTGA
*hsp-16.21*	AGATATGGGCGGAATGCAAC	GACTTTCAGCTCTTCTGGCTTG

## Data Availability

The RNA sequencing data generated in this study have been deposited in the NCBI Sequence Read Archive (SRA) under the BioProject accession number PRJNA1357865. The data are publicly available at: https://www.ncbi.nlm.nih.gov/bioproject/PRJNA1357865 (accessed on 16 November 2025).

## Supplementary Materials

The following supporting information can be downloaded at: https://www.mdpi.com/article/10.3390/antiox14121390/s1, Table S1: Lifespan statistics of N2 *C. elegans* treated with different concentrations of GOP; Table S2: Differentially expressed genes between GOP400-treated and control N2 *C. elegans*.
