# Supplementary material for: Ginseng Oligopeptides Promote Longevity and Enhance Stress Resistance in Caenorhabditis elegans via the DAF-16/FOXO Pathway
Source: Antioxidants (Basel). 2025 Nov 21;14(12):1390. doi: 10.3390/antiox14121390 (PMC12729657; doi:10.3390/antiox14121390)
Supplement: Supplementary file 1 [file antioxidants-14-01390-s001.zip › Table S1 Lifespan statistics of N2 C. elegans treated with different concentrations of GOP.docx]

**Table S1. Lifespan statistics of N2 *C. elegans* treated with different concentrations of GOP.**

Data represent results from three independent lifespan assays (n = 60 worms per group). Median and maximum lifespans were calculated using Kaplan–Meier survival analysis. Log-rank (Mantel–Cox) tests were applied to assess statistical significance between each treatment and the control group.

| **Group** | **N** | **Deaths** | **Censored** | **Min(day)** | **Max(day)** | **Mean ± SE(day)** | **Median(day)** | **Logrank *χ^2^*** | ***p*_value** |
| --- | --- | --- | --- | --- | --- | --- | --- | --- | --- |
| Control | 60 | 39 | 21 | 1 | 34 | 23.03 ± 1.32 | 26 |  |  |
| GOP100 | 60 | 42 | 18 | 1 | 33 | 23.38 ± 0.97 | 26 | 0.843713975 | 0.358336728 |
| GOP200 | 60 | 38 | 22 | 1 | 35 | 23.97 ± 1.24 | 29 | 2.436211479 | 0.118562265 |
| GOP400 | 60 | 46 | 14 | 1 | 36 | 27.35 ± 1.10 | 29 | 7.588417905 | 0.005874448 |
